# Plant-Growth-Promoting Rhizobacteria Improve Seeds Germination and Growth of *Argania spinosa*

**DOI:** 10.3390/plants13152025

**Published:** 2024-07-24

**Authors:** Naima Chabbi, Salahddine Chafiki, Maryem Telmoudi, Said Labbassi, Rachid Bouharroud, Abdelghani Tahiri, Rachid Mentag, Majda El Amri, Khadija Bendiab, Driss Hsissou, Abdelaziz Mimouni, Naima Ait Aabd, Redouan Qessaoui

**Affiliations:** 1 Regional Center of Agricultural Research of Agadir, National Institute of Agricultural Research (INRA), Avenue Ennasr, BP415 Rabat Principale, Rabat 10090, Morocco; 2 Laboratory of Agrobiotechnology and Bioengineering, Department of Biology, Faculty of Science and Technology-Gueliz, Cadi Ayyad University, Marrakesh 4000, Morocco; 3 AgroBioSciences Department, Mohammed VI Polytechnic University, Lot 660, Hay Moulay Rachid, Ben Guerir 43150, Morocco; 4 Biotechnology Unit, Regional Center of Agronomic Research of Rabat, National Institute of Agricultural Research, Avenue Ennasr, BP415 Rabat Principale, Rabat 10090, Morocco

**Keywords:** *Argania spinosa*, PGPR, seedling, plant growth, biofertilization

## Abstract

*Argania spinosa* is among the most important species of the Moroccan forest in terms of ecological, environmental, and socio-economic aspects. However, it faces a delicate balance between regeneration and degradation in its natural habitat. Hence, the efforts to preserve and regenerate argan forests are crucial for biodiversity, soil quality, and local livelihoods, yet they face challenges like overgrazing and climate change. Sustainable management practices, including reforestation and community engagement, are vital for mitigating degradation. Similarly, exploiting the argan tree’s rhizosphere can enhance soil quality by leveraging its rich microbial diversity. This approach not only improves crop growth but also maintains ecosystem balance, ultimately benefiting both agriculture and the environment. This enrichment can be achieved by different factors: mycorrhizae, plant extracts, algae extracts, and plant growth-promoting rhizobacteria (PGPR). The benefits provided by PGPR may include increased nutrient availability, phytohormone production, shoot, root development, protection against several plant pathogens, and disease reduction. In this study, the effect of rhizobacteria isolated from the Agran rhizosphere was evaluated on germination percentage and radicle length for *Argania spinosa* in vitro tests, growth, collar diameter, and branching number under greenhouse conditions. One hundred and twenty (120) bacteria were isolated from the argan rhizosphere and evaluated for their capacity for phosphate solubilization and indole acetic acid production. The results showed that 52 isolates could solubilize phosphorus, with the diameters of the solubilization halos varying from 0.56 ± 0.14 to 2.9 ± 0.08 cm. Among 52 isolates, 25 were found to be positive for indole acetic acid production. These 25 isolates were first tested on maize growth to select the most performant ones. The results showed that 14 isolates from 25 tested stimulated maize growth significantly, and 3 of them by 28% (CN005, CN006, and CN009) compared to the control. Eight isolates (CN005, CN006, CN004, CN007, CN008, CN009, CN010, and CN011) that showed plant growth of more than 19% were selected to evaluate their effect on argan germination rate and radicle length and were subjected to DNA extraction and conventional Sanger sequencing. The 8 selected isolates were identified as: *Brevundimonas naejangsanensis* sp2, *Alcaligenes faecalis*, *Brevundimonas naejangsanensis* sp3, *Brevundimonas naejangsanensis* sp4, *Leucobacter aridicollis* sp1, *Leucobacter aridicollis* sp2, *Brevundimonas naejangsanensis* sp1, and *Staphylococcus saprophyticus*. The results showed that *Leucobacter aridicollis* sp2 significantly increased the germination rate by 95.83%, and the radicle length with a value of 2.71 cm compared to the control (1.60 cm), followed by *Brevundimonas naejangsanensis* sp3 and *Leucobacter aridicollis* sp1 (2.42 cm and 2.11 cm, respectively). Under greenhouse conditions, the results showed that the height growth increased significantly for *Leucobacter aridicollis* sp1 (42.07%) and *Leucobacter aridicollis* sp2 (39.99%). The isolates *Brevundimonas naejangsanensis* sp3 and *Leucobacter aridicollis* sp1 increased the gain of collar diameter by 41.56 and 41.21%, respectively, followed by *Leucobacter aridicollis* sp2 and *Staphyloccocus saprophyticus* (38.68 and 22.79%). *Leucobacter aridicollis* sp1 increased the ramification number per plant to 12 compared to the control, which had 6 ramifications per plant. The use of these isolates represents a viable alternative in sustainable agriculture by improving the germination rate and root development of the argan tree, as well as its development, while increasing the availability of nutrients in the soil and consequently improving fertilization.

## 1. Introduction

The argan tree (*Argania spinosa*) is one of the only trees of the tropical family *Sapotaceae* identified in the Moroccan vegetation. This crop is one of the most important species of the Moroccan forest in ecological, environmental, and socio-economic terms. *A. spinosa* is known for its ecological role, which is to stabilize soils and protect the soil from erosion due to its deep and powerful roots, consequently slowing down aridification processes, while also shading other crops and maintaining soil fertility [1]. From the economic side, the argan tree’s various components, including wood, leaves, fruits, and oil, can be exploited as sources of income or food. Argan oil is very valuable and used not only for food consumption but also for cosmetic and medicinal purposes, and it has been proposed as a nutraceutical. The leaves and fruits of the argan tree are exploited as hanging forage, while the wood is used as fuel. However, the argan tree is facing very important regressive dynamics due to the combined effects of the aridity of the climate, the scarcity and irregularity of rainfall, and human pressure [2]. On the other hand, the regeneration of the argan tree is practically absent in the main area of distribution of this species [3]. A rescue intervention and rapid safeguarding of the argan groves are necessary.

Nevertheless, the water deficit and poverty of the soil considerably reduce the success of the plantations. Its cultivation or domestication will lead to important economic opportunities for Morocco [4], given the growing demand for argan oil and its high price. Furthermore, *A. spinosa* exhibits slow growth rates and has evolved to thrive in arid environments. These trees have developed specific leaf traits and physiological adaptations to survive and grow in harsh conditions where few other tree species can survive [5].

The green revolution in agricultural production has increased the use of agrochemical products (pesticides, herbicides, and fertilizers) to increase crop productivity and maintain food security, which is having a deleterious effect on our environment and health [6].

Exploiting the rhizospheric soils of different plants has been very important for soil enrichment (biofertilization) and the better development of several crops. This enrichment can be achieved by various mechanisms, such as mycorrhizae [7], plant extracts, algae extracts, and PGPR [7,8]. Rhizosphere bacteria can enhance plant growth through a wide variety of mechanisms, mainly phosphate solubilization, siderophore production [9], biological nitrogen fixation, the production of 1-aminocyclopropane-1-carboxylate deaminase (ACC), and phytohormone production (Auxins, GAs, ABA, and CKs) [8,10]. Additionally, PGPR can emit volatile organic compounds (VOCs) such as acetoin, 2,3-butanediol, and hydrogen cyanide, which can inhibit fungal growth either directly or indirectly by modulating plant defense responses [11,12]. The beneficial effects of PGPR on plants also include stimulation of seed germination [13] and plant development, as well as enhancement of mineral elements and water use [13]. Ref. [14] underscored the vital role of argan rhizospheric soil in facilitating alfalfa germination and growth, even in harsh conditions like drought and salinity, through the expedited and enriched germination process observed with normal irrigation using rhizospheric filtrates. While research on plant growth-promoting rhizobacteria (PGPR) in argan trees remains limited, studies have revealed [15] significant microbial diversity in both the root system and residues, comprising beneficial species like nitrogen-fixing organisms and organic matter decomposers. Ref. [16] isolated *Streptomyces marokkonensis* sp. from argan tree rhizosphere soil, known for its production of the inhibitory compound isochainin against pathogenic yeasts and fungi, and [17] found that two *Streptomyces* strains caused a significant increase in tomato root and shoot lengths, dry/fresh weights, and vigor index values compared to the control. Refs. [18,19] identified polyhydroxybutyrate-producing bacteria, including *Serratia*, *Sphingomonas*, and *Proteus*, in agricultural soil samples from the argan tree’s southern region, which are detected as biofertilizants and biostimulants by auxin production, nitrogen fixation, and cytokinin production [20,21]. Furthermore, [22] demonstrated superior amylase activity in bacteria isolated from argan tree rhizospheric soil compared to other soil types. Ref. [15] highlighted the importance of microbial diversity associated with argan tree roots and residues, with *Actinobacteria* and *Proteobacteria*, being identified as potential plant growth-promoting rhizobacteria (PGPR) in the context of argan tree ecosystems, emerging as the most significant taxonomic groups, as evidenced by their elevated operational taxonomic units at 97% of identity levels. These findings collectively underscore the crucial role of microorganisms in supporting the health and resilience of argan trees in their arid environment.

Furthermore, this study aimed to evaluate the effect of rhizospheric bacteria on argan seed germination and seedling growth. To achieve this objective, a series of experiments was conducted to evaluate the rhizobacteria’s ability to solubilize phosphorus and produce auxin, as well as their potential to enhance maize growth. Subsequently, the strains demonstrating proficiency in phosphorus solubilization, auxin production, and maize growth enhancement were selected for inoculating argan seeds to investigate their capacity to stimulate germination and promote the growth of *Argania spinosa* seedlings. To achieve these objectives, the study was designed to understand how rhizospheric bacteria impact vital aspects of Argan seedling development and to elucidate the potential mechanisms and relationships between bacterial traits and seedling growth.

## 2. Materials and Methods

### 2.1. Soil Sample Collection

To isolate plant growth-stimulating bacteria, 3 soil samples were collected in the rhizosphere argan tree at 20–30 cm depth [15], in 3 different regions of Morocco: Belfaa region (30°01′35″ N 9°33′15″ W), Chtouka Ait Baha Province, Ait Meloul in Souss-Massa region (30°21′03″ N 9°30′25″ W), and Laayoune-Sakai El Hamra region (25°26′36.924″ N 13°10′26.526″ W).

### 2.2. Bacteria Isolation

Isolation of rhizospheric bacteria was carried out by taking 1 g of soil adherent to the roots for each sample and adding 9 mL of sterile physiological water and shaking for 5 min, then a series of dilutions (10^−3^ to 10^−8^) was made. 0.1 mL of each dilution was spread on a Petri dish containing nutrient agar (NG). The Petri dishes were incubated at 28 °C for 48 h. The different colonies were pricked out and transferred to a new Petri dish containing GN to obtain pure cultures. A total of 120 isolates were replicated. The isolates were stored at 4 °C on yeast dextrose carbonate (YDC) agar (Sigma-Aldrich Chemical Co., St. Louis, MO, USA) for a short period and at −80 °C in 40% glycerol [23] for long-term storage.

### 2.3. Screening for Phosphate Solubilization

The isolates were tested for their capacity to solubilize phosphate using the National Botanical Research Institute’s Phosphorus (NBRIP) solid medium [24]. The pH of the NBRIP media was adjusted to 6.75 ± 0.25 before autoclaving. A volume of 10 µL of each bacterial culture was dropped on the surface of the NBRIP medium. After 7 days of incubation at 30 °C, the diameter of the halo around the colony was measured. A positive result of phosphate solubilization was indicated with a transparent halo around the colony [25].

### 2.4. Screening for Indole Acetic Acid (IAA) Production

The capacity of IAA production by bacterial isolates was tested following the method described by [26]. 10 µL of each bacterial culture was grown in Luria–Bertani (LB) solid medium supplemented with 5 mM of tryptophan (Sigma-Aldrich Chemical Co., St. Louis, MO, USA) and incubated at 27 °C for 3 days. Then, 1 mL of Salkowski’s reagent (2% 0.5 M FeCl_3_ in 35% perchloric acid) was saturated into the paper. The presence of IAA in the medium was confirmed by a reddish-to-pink coloration formed around the colony.

### 2.5. Effect of Isolate on Maize Growth

Maize seeds were sterilized in 1% sodium hypochlorite for 10 min, followed by three washes with sterile water. Seeds were inoculated by immersion for 1 h in the inoculum solution (10^8^ CFU/mL) with 1% xanthan gum [27]. After inoculation, the seedlings were transplanted into plastic pots containing 100% peat. Pots were placed in the greenhouse (T˚ = 25 ± 2 °C, RH = 70 ± 5%). We used a completely randomized block design with (n = 30) seeds for each treatment. After 45 days, the plant height was measured.

### 2.6. In Vitro Effect of the Isolates on the Growth of Argan Seedlings

The isolates that showed a significant effect on maize growth were tested on the seed germination of *A. spinosa* in vitro.

Seeds collected from the same plant were first broken to remove the shell. The kernels were sterilized on the surface with a solution of sodium hypochlorite (2%) for 10 min, rinsed three times with sterile distilled water, and dried in septic conditions. The kernels were inoculated with a bacterial suspension (10^8^ CFU/mL) combined with 1% xanthan. Control seeds were treated with sterile distilled water combined with 1%xanthan. Treated and control seeds were placed in 9 cm Petri dishes containing filter paper immersed with sterile distilled water (1 mL) with 24 kernels for each treatment and 8 kernels in each Petri dish. The plates were incubated for 7 days at 20 °C. The germination rate and the radicle length were measured.

The germination rate was calculated using the following formula:

$$Germination\;rate\;(\%)=\frac{Number\;of\;germinated\;kernels}{Total\;number\;of\;kernels}\times 100$$

### 2.7. In Vivo Effect of PGPR Isolates on the Growth of Argan Seedlings

One-and-a-half-year-old argan seedlings were transplanted into 5L pots containing a mixture of sand and peat (2:1 (*v*/*v*)). The soil mixture was pre-mixed with a bacterial suspension (10^8^ CFU/g). The plants were placed in a greenhouse (25 ± 2 °C and an RH of 70 ± 5%) and irrigated twice weekly. Each treatment consisted of 17 plants, and a completed randomized block design was applied. Plant height (%), collar diameter (%), and number of ramifications were evaluated.

$$Plant\;height\;gain\left(\%\right)=\frac{Hf-Hi}{Hf}\times 100$$

(1) $$Collar\;diameter\;gain\left(\%\right)=\frac{Df-Di}{Df}\times 100$$

i: initial measures
f: final measures

The isolates that showed a significant effect on *A. spinosa* seedlings were selected for analysis of their PGPR properties.

### 2.8. Available Phosphate Production (P_2_O_5_)

The available phosphate production (P_2_O_5_) was quantitatively estimated in Erlenmeyer flasks (100 mL) containing 50 mL of NBRIP medium. The flasks were inoculated with 100 µL of cell suspension (10^8^ CFU/mL) and incubated at 28 °C for 7 days at 120 rpm. The concentration of P_2_O_5_ in the culture supernatant was estimated using the method described by Olsen [28]. Three replicates were used for each isolate using a visible spectrophotometer, ONDA v-10 plus (Beijing, China).

### 2.9. Indole Acetic Acid (IAA) Production

IAA production was determined using a colorimetric method developed by [26]. 100 µL of bacterial culture containing 10^8^ CFU/mL was added to 100 mL of Luria–Bertani (LB) medium supplemented with 5 mM L-tryptophan (Sigma-Aldrich Chemical Co., St. Louis, MO, USA). The inoculated medium was incubated at 28 °C with agitation for 48 h. After the incubation period, 50 mL of bacterial culture was centrifuged at 13,000 rpm for 10 min. Then, 1 mL of each supernatant was mixed with 2 mL of Salkowski reagent (2% 0.5 M FeCl_3_ in 35% perchloric acid) and incubated at room temperature for 20 min. The optical density was then measured at 535 nm using a visible spectrophotometer, ONDA v-10 plus (Beijing, China).

### 2.10. Nitrogen Fixation

The nitrogen-fixing ability of selected isolates was tested using Burk’s N-free medium [29,30,31]. All isolates were streaked on nitrogen-free media and incubated at 37 °C for 7 days. Only bacteria capable of fixing atmospheric nitrogen will grow in this nitrogen-free medium.

### 2.11. Hydrogen Cyanide Production

The potential of the isolates to produce hydrogen cyanide was evaluated using the method described by Lorck [32]. The isolates were stained on a nutrient agar amended with 4.4 g/L glycine (Sigma-Aldrich Chemical Co., St. Louis, MO, USA). A sterile Whatman filter paper was saturated in a 0.5% solution of picric acid (Sigma-Aldrich Chemical Co., St. Louis, MO, USA) and placed inside the lid of a cultured Petri dish. Lids were covered using parafilm and incubated for 48 h at 25 °C. The synthesis of HCN was detected by the change of the Whatman paper color from yellow to orange or brown [12].

### 2.12. Siderophores Production

A modified CAS agar medium was used to detect the ability of the isolates to produce siderophores [33]. CAS agar plates were prepared by mixing 100 mL of CAS reagent with 900 mL of sterilized LB agar medium. The CAS reagent was prepared by dissolving 121 mg of CAS (Sigma-Aldrich Chemical Co., St. Louis, MO, USA) in 100 mL of distilled water and 20 mL of a 1 mM ferric chloride (FeCl_3_·6H_2_O) solution prepared in 10 mM HCl. This solution was added to a 20 mL hexadecyl trimethyl ammonium bromide (HDTMA) solution. The HDTMA solution was prepared by mixing 729 mg of HDTMA with 400 mL of distilled water [33] The CAS–HDTMA solution was sterilized before further use. After inoculation, the plates were incubated at 28 °C for 5 days and observed for the formation of an orange zone around the bacterial colonies, and an un-inoculated plate was used as a control [9].

### 2.13. Ammonia Production

Ammonia production was evaluated using Nessler’s reagent [34]. The isolates were grown in peptone broth for 48 h at 25 °C. After centrifugation (1000 rpm), 1 mL of supernatant was added to 1 mL of Nessler’s reagent. The appearance of a brown-to-yellow color is an indication of ammonia production.

### 2.14. Bacterial Characterization

Four selected bacteria were grown on GN medium for 24 h at 28 °C, the color and shape of the colonies were observed, and Gram staining was performed.

The strains were then characterized for the following biochemical traits: catalase and oxidase. The physiological traits of the samples were characterized, including pH, temperature, and NaCl conditions.

### 2.15. DNA Extraction, 16S rRNA Sequencing, and Analysis

The total genomic DNA from the different strains was extracted from overnight cultures grown in GN medium. DNA extraction of bacteria was conducted using the Higher Purity™ Bacterial Genomic DNA Isolation Kit (Canvax Biotech, S.L., Cordoba, Spain), according to the manufacturer’s instructions. The quality and concentration of DNA extracts were assessed by determination of absorbance at 260 nm and 280 nm (BioDrop µLite^+^ Micro volume Spectrophotometer, Cambridge, MA, USA)

To assess the molecular identities of the 8 strains, their 16S rRNA genes were amplified by PCR using the universal primers PA (5′-AGAGTTTGATCCTGCTCAG-3′) and PH (5′-AAGGAGGTGATCCAGCCGCA-3′) described previously [35], PCR was performed on 150 ng DNA, with 0.5 U KAPA2G fast DNA polymerase (KAPA Biosystems, Wilmington, MA, USA), 1× KAPA2G buffer, 1.5 mM MgCl_2_, 0.2 mM each dNTP, and 300 nM each primer in a 25 µL reaction volume under the following conditions: preheating at 95 °C for 2 min, then 35 cycles of denaturation at 95 °C for 15 s, annealing at 60 °C for 15 s and extension at 72 °C for 2 s, followed by a final extension of 30 s at 72 °C. The amplified products were purified using EXOSAP-IT (Affymetrix), and sequence reactions were performed with the primer PEF (5′-CATGGCTGTCGTCAGCTCGT-3′) [35] using a BigDye terminator cycle sequencing kit version 3.1 (Applied Biosystems, Waltham, MA, USA). Sequencing products were purified using G50 gel filtration (Sephadex G50 superfine; Sigma Aldrich, St. Louis, MO, USA) and loaded onto an ABI3130XL capillary sequencer. The produced electropherograms were analyzed by the sequencing analysis software version 5.3.1 (Applied Biosystems).

The nucleotide sequences were submitted to the NCBI database (National Center for Biotechnology Information, Bethesda, MD, USA) (https://www.ncbi.nlm.nih.gov, accessed on 21 May 2024) and analyzed with BLASTn available online (https://blast.ncbi.nlm.nih.gov/Blast.cgi, accessed on 21 May 2024) to identify sequences of interest by comparing them with known sequences in the search tool. The generated sequences from this study were deposited in the NCBI database. The maximum likelihood (ML) approach with heuristic searches was used to construct a phylogenetic tree, with all other parameters set to their default values. The bootstrap method with 1000 replicates was employed to assess the robustness of the clades. As the outgroup, the sequence of *Sulfolobus solfataricus* belonging to the family Sulfolobaceae (X03235) was used.

### 2.16. Statistical Analysis

Data were subjected to ANOVA using the R Studio program (Posit Software, PBC, 2024.04.1-748). Data for the growth of *A. spinosa* seedlings are presented as means with a standard deviation. Any difference mentioned is significant at *p* < 0.05 using the Tukey test.

## 3. Results

### 3.1. Screening for Phosphorate Solubilization

**Figure 1:**
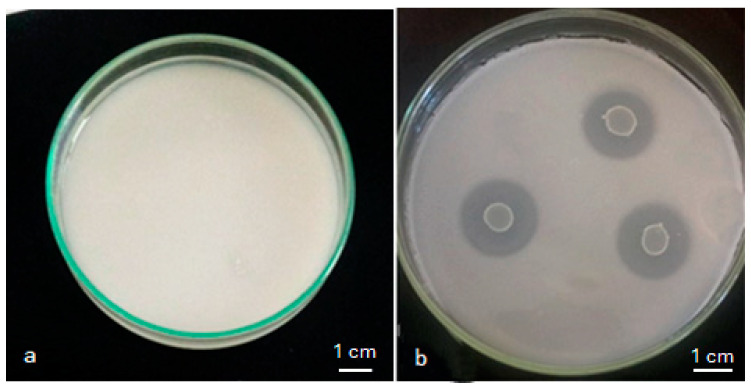
Phosphate solubilization in NBRIP medium: (**a**) absence of the halo; (**b**) presence of the halo.

The results showed that, among 120 isolates, 52 could solubilize phosphate. The solubilization was manifested by a clear halo around the colonies in the medium (Figure 1). Furthermore, the halo of solubilization varied from 0.56 ± 0.14 cm to 2.90 ± 0.08 cm for isolates CN003 and CN010, respectively (Table 1).

### 3.2. Screening for Indole Acetic Acid (IAA) Production

**Figure 2:**
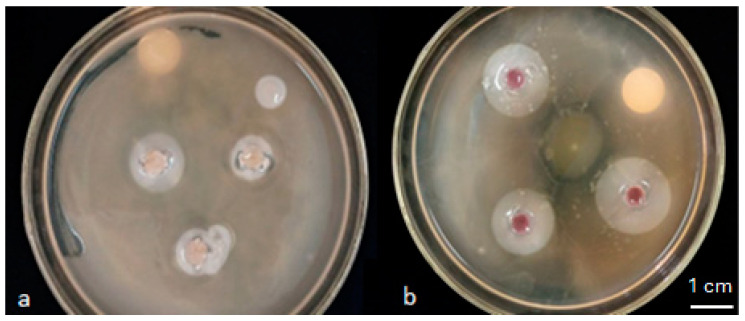
Indole Acetic Acid (IAA) production; (**a**): non-auxin-producing bacteria; (**b**): auxin-producing bacteria.

The 52 isolates that showed phosphate solubilization were tested for their IAA production. The results demonstrated that among the 52 bacteria tested, 25 were able to produce IAA in the LBT medium. The IAA production was manifested by a reddish-to-pink coloration formed around the colony after adding the Salkowski reagent (Figure 2).

### 3.3. Growth-Promotion Experiments

#### 3.3.1. In Vitro Effect of the Isolates on the Growth of Maize Seedlings

**Figure 3:**
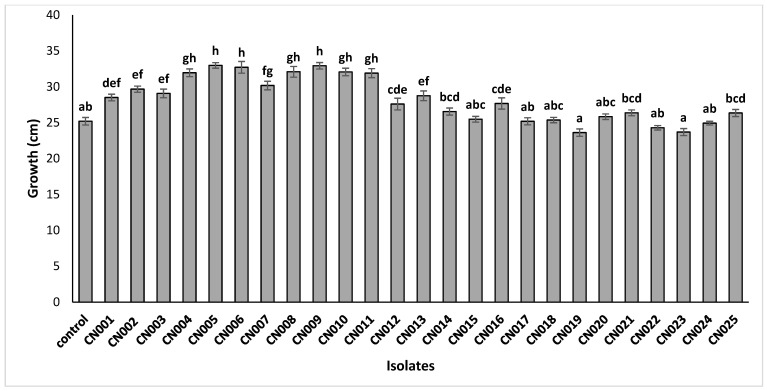
Effect of bacterial isolates on the growth of maize seedlings. The bars with the same letters are not significantly different at a 5% significance level, according to the Tukey test.

The 25 isolates that showed phosphorate solubilization and IAA production were selected to evaluate their growth promotion effect on maize seedlings. The results showed that among the 25 isolates tested, 20 showed a positive effect on maize growth. Compared to the uninoculated plants, this growth gain varied from 26.36 cm to 32.97 cm, as shown by CN021 and CN005 isolates, respectively. Statistical analysis showed that the isolates (CN004, CN005, CN006, CN008, CN009, CN010, and CN011) showed a highly significant effect on the growth gain of maize plants compared with others (Figure 3).

The eight isolates (CN004, CN005, CN006, CN007, CN008, CN009, CN010, and CN011) exhibiting a growth exceeding 30 cm were chosen for further in vitro assessment on argan seeds to evaluate their germination rate and radicle length. The isolates were then subjected to DNA extraction.

#### 3.3.2. Isolates Effect on the *Argania spinosa* Seeds In Vitro Germination

**Figure 4:**
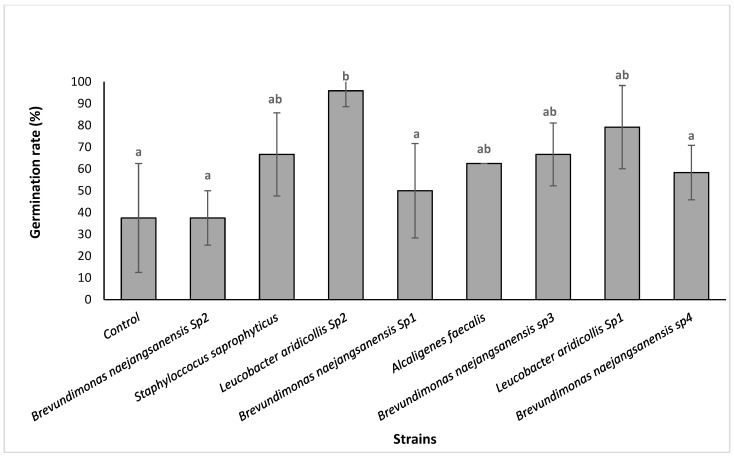
Bacterial effect on the in vitro germination rate of argan seeds. The bars with the same letters are not significantly different at a 5% significance level, according to the Tukey test.

**Figure 5:**
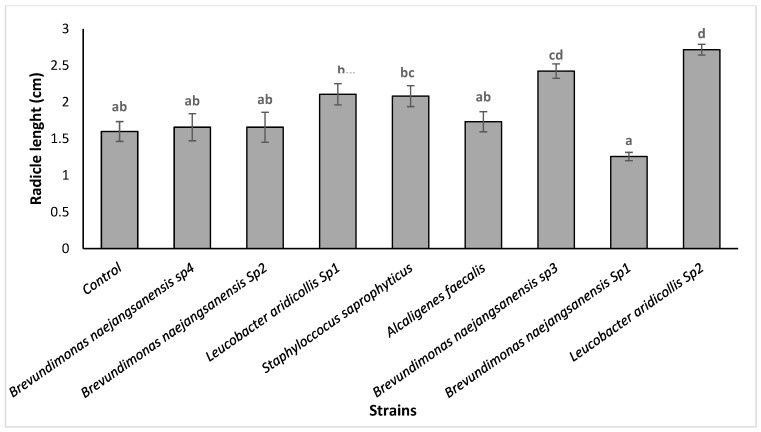
Effect of bacterial isolates on radicle length gain of argan seeds in vitro. The bars with the same letters are not significantly different at a 5% significance level, according to the Tukey test.

**Figure 6:**
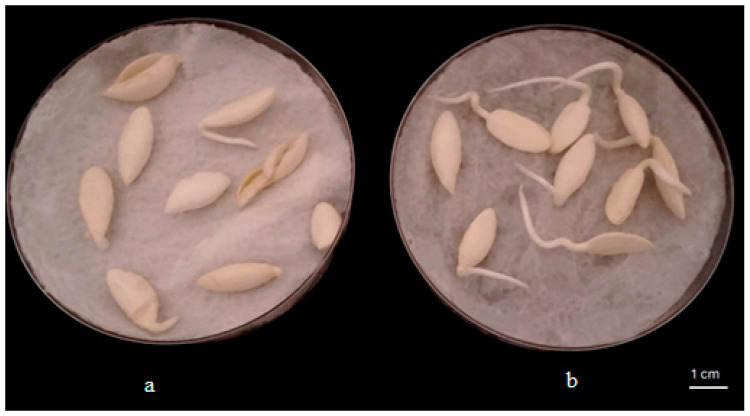
Effect of bacterial strains on seed germination of *Argania spinosa* in vitro after 72 h of incubation in the dark at 25 °C: (**a**) uninoculated seeds; (**b**) inoculated with plant-growth-promoting rhizobacteria *Leucobacter aridicollis* sp1.

The results showed that the isolates (*Staphyloccocus saprophyticus*, *Leucobacter aridicollis* sp2, *Brevundimonas naejangsanensis* sp1, *Alcaligenes faecalis*, *Brevundimonas naejangsanensis* sp3, and *Leucobacter aridicollis* sp1) showed a stimulating effect on seed germination ranging from 37.5 to 95.8%. The statistical analysis showed that the isolate *Leucobacter aridicollis* sp2 significantly increased germination by 95.83% compared to the control (37.5%) (Figure 4). The results showed that the isolates significantly improved the radicle length, ranging from 2.10 cm to 2.71 cm, with the greatest improvement for the *Leucobacter aridicollis* sp1 (2.71 cm) and *Brevundimonas naejangsanensis* sp3 (2.42 cm), respectively (Figure 5 and Figure 6).

Four strains (*Staphyloccocus saprophyticus*, *Leucobacter aridicollis* sp2, *Brevundimonas naejangsanensis* sp3, and *Leucobacter aridicollis* sp1), showing a significant in vitro effect on germination rate and radicle length gain, were selected to evaluate their effects on argan seedling growth under greenhouse conditions.

#### 3.3.3. Effect of the Isolates on the Growth of *Argania spinosa* Seedlings under Greenhouse Conditions

**Figure 7:**
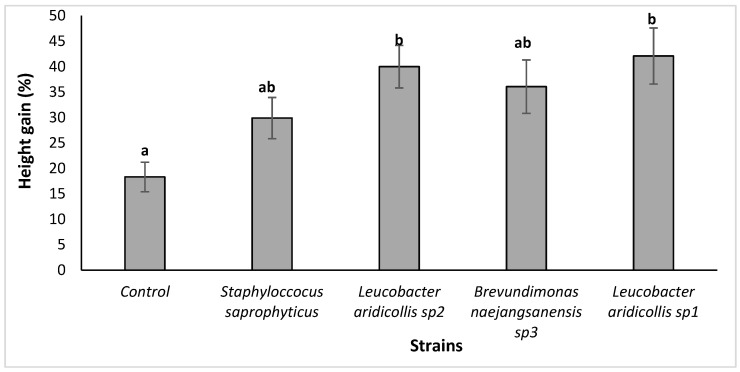
Effect of bacterial isolates on height growth of *Argania spinosa* seedlings under greenhouse conditions. The bars with the same letters are not significantly different at a 5% significance level, according to the Tukey test.

**Figure 8:**
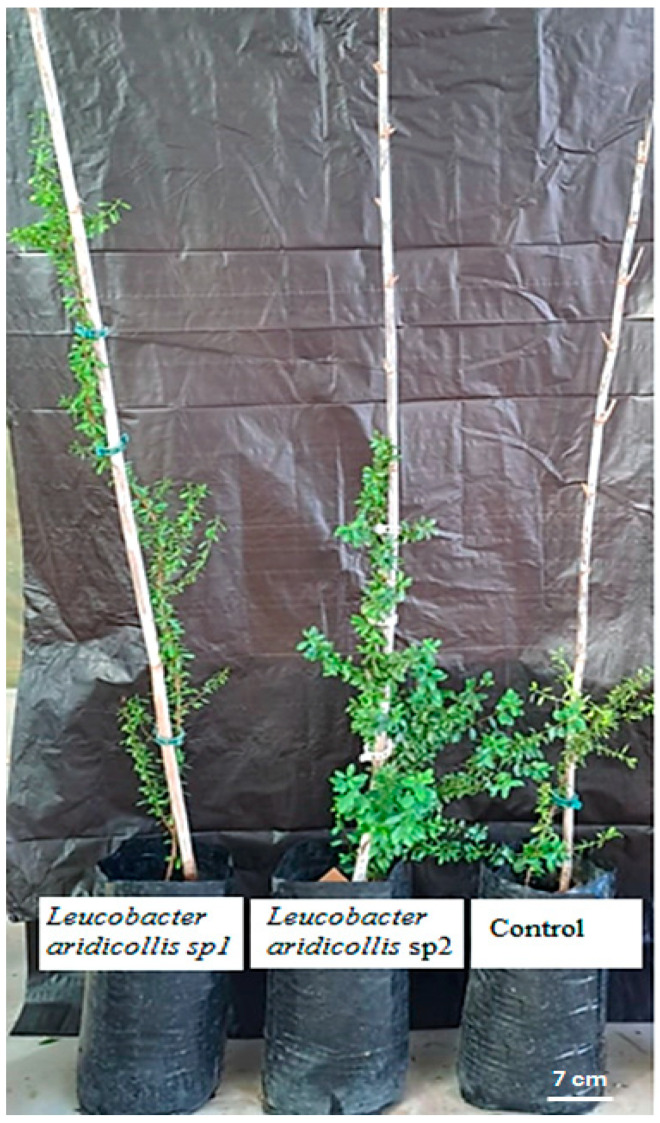
Bacterial strains’ effect on *Argania spinosa* seedlings growth.

**Figure 9:**
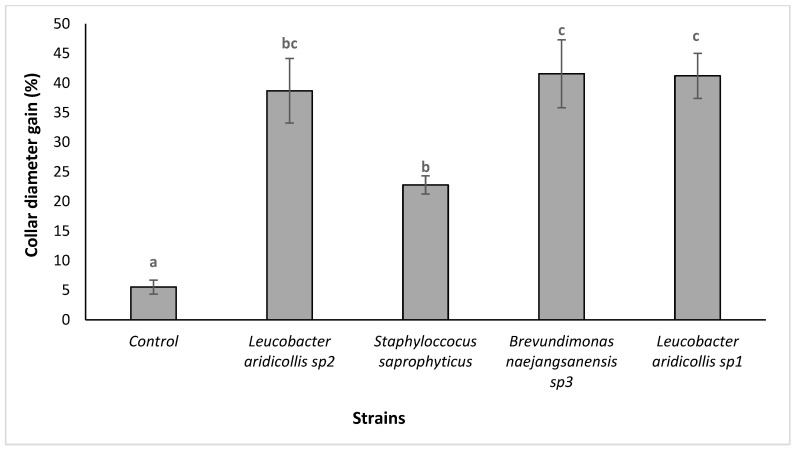
Effect of bacterial strains on the collar diameter of *Argania spinosa* seedlings under greenhouse conditions. The bars with the same letters are not significantly different at a 5% significance level, according to the Tukey test.

The results showed that the isolates of *Leucobacter aridicollis* sp2 and *Leucobacter aridicollis* sp1 increased plant height significantly by 39.99 and 42.07%, respectively (Figure 7 and Figure 8). The four tested isolates (*Brevundimonas naejangsanensis* sp3, *Leucobacter aridicollis* sp1, *Leucobacter aridicollis* sp2, and *Staphyloccocus saprophyticus*) significantly increased the collar diameter. The most important isolates were *Brevundimonas naejangsanensis* sp3 and *Leucobacter aridicollis* sp1, with 41.56 and 41.21%, respectively, followed by *Leucobacter aridicollis* sp2 (38.68%) and *Staphyloccocus saprophyticus* (22.79%) (Figure 9).

**Figure 10:**
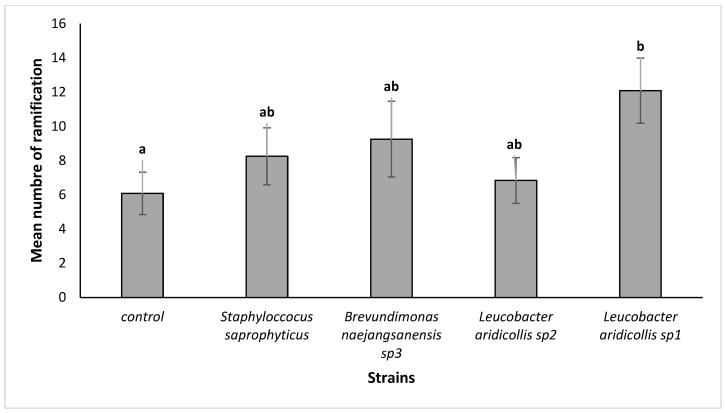
Effect of bacterial strains on branching out of *Argania spinosa* seedlings under greenhouse conditions. The bars with the same letters are not significantly different at a 5% significance level, according to the Tukey test.

**Figure 11:**
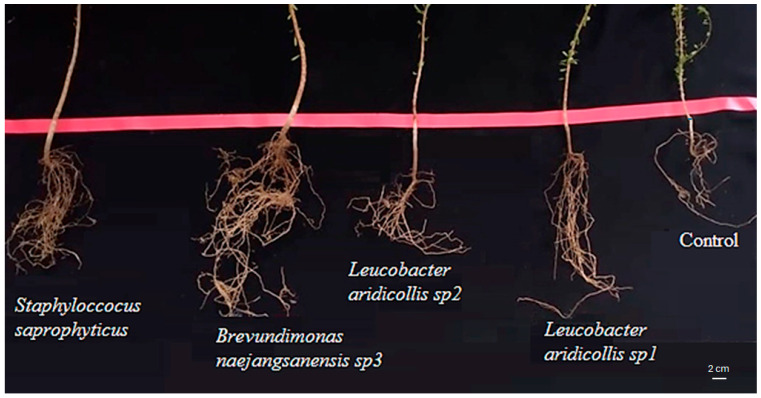
Bacterial strain’s effect on the root growth of *Argania spinosa* seedlings.

The isolate *Leucobacter aridicollis* sp1 significantly improves the ramification number to 12.00 (Figure 10) compared to the control (6.00 ramification/plant). Those plant growth-promoting rhizobacteria also influenced the length and density of argan roots. The largest root system was observed in the plant inoculated with *Brevundimonas naejangsanensis* sp3 compared to the control (Figure 11).

### 3.4. PGPR Parameters

Four selected strains (*Staphyloccocus saprophyticus*, *Brevundimonas naejangsanensis* sp3, *Leucobacter aridicollis* sp2, and *Leucobacter aridicollis* sp1) that demonstrated a significant impact on the growth of argan were assessed for their PGPR parameters, including nitrogen fixation, ammonia production, siderophore production, IAA, and available phosphate (P_2_O_5_).

The results showed that the four isolates produced a high amount of available phosphate (ranging from 36.21 ± 0.11 to 49.11 ± 0.76 µg/mL for *Brevundimonas naejangsanensis* sp3 and *Staphyloccocus saprophyticus*, respectively) and IAA (ranging from 27.83 ± 0.49 to 137.14 ± 0.33 µg/mL for *Brevundimonas naejangsanensis* sp3 and *Staphyloccocus saprophyticus*, respectively) (Table 2). The strain *Staphyloccocus saprophyticus* exhibited a high capacity for producing IAA and available phosphate, followed by *Leucobacter aridicollis* sp2 (Table 2). The results also demonstrated that all four strains produced ammonia and siderophores. The siderophore production is manifested by the formation of an orange zone around the bacterial colonies, while the ammonia production is indicated by the appearance of a brown-to-yellow color. Furthermore, only *Leucobacter aridicollis* sp1 displayed nitrogen fixation capabilities, and strain *Leucobacter aridicollis* sp2 was the only one to demonstrate HCN production (Table 2).

For morphological, biochemical, and physiological characterization, the bacterial isolates demonstrated distinct characteristics. *Staphylococcus saprophyticus* differed as a cocci, unlike the other bacilli isolates. While all the isolates were Gram-positive, *Staphylococcus saprophyticus* was Gram-negative. In the catalase test, all isolates were positive, except for *Staphylococcus saprophyticus*, which showed a negative result, unlike the rest. Motility tests yielded negative results for all isolates.

Regarding environmental resilience, all isolates tolerated a pH range of 4 to 12. However, *Leucobacter aridicollis* sp1 could not withstand a NaCl concentration of 40 g/L, unlike others, which thrived across NaCl concentrations ranging from 5 g/L to 40 g/L. Additionally, while all isolates grew at 30 °C, only *Leucobacter aridicollis* sp2 and *Brevundimonas naejangsanensis* sp3 grew at 4 °C, and *Staphylococcus saprophyticus* showed growth even at 55 °C (Table 3).

### 3.5. 16S rRNA Sequencing and Analysis of the Eight Strains

The nucleotide sequences of the PCR-amplified products were determined using Sanger sequencing, yielding a final fragment ranging from 458 to 560 bp. The BLASTn comparison in the NCBI GenBank database search revealed that strain CN011 shared 100% of similarities with reported sequences from *Staphylococcus saprophyticus* deposited in NCBI under accession numbers NR_074999. The results indicated also that the sequences of strains CN006, CN004, CN010, and CN007 had more than 99% sequence homology to the *Brevundimonas naejangsanensis* nucleotide sequence contained in the NCBI database under accession number NR_116722. Furthermore, strains CN009 and CN008 have shared 100% sequence similarities with the reported nucleotide sequence from *Leucobacter aridicollis* under accession number NR_042288. While the strain CN005 nucleotide sequences shared 100% of similarities with reported sequences from *Alcaligenes faecalis* deposited in NCBI under accession number NR_043445. The 16s rRNA gene sequences of the eight bacterial isolates were submitted to the NCBI GenBank nucleotide database under the following accession numbers: PP823857 (strain CN004), PP829041 (strain CN005), PP823858 (strain CN006), PP823859 (strain CN007), PP823860 (strain CN008), PP823861 (strain CN009), PP823862 (strain CN010), and PP823863 (CN011).

**Figure 12:**
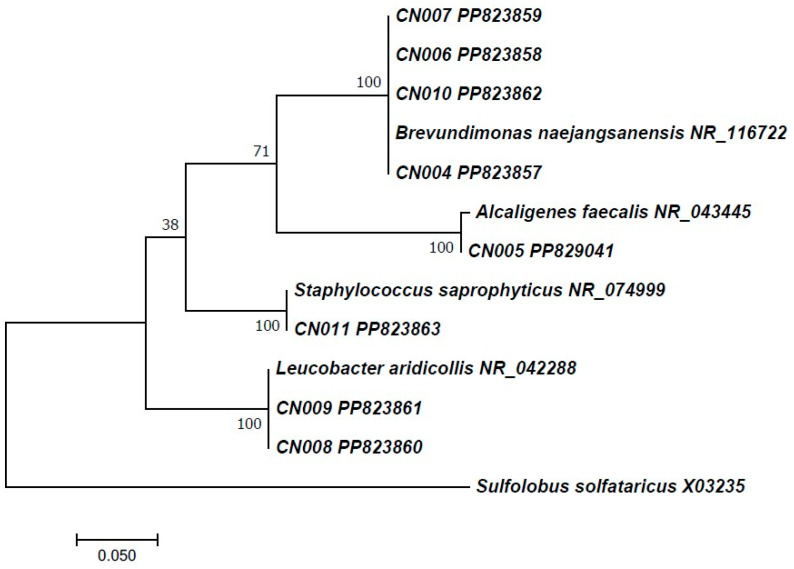
Phylogenetic tree constructed according to the Maximum Likelihood method using gene sequences of 16S rRNA regions (PEF). The numbers at branches indicate the bootstrap support values (expressed as percentages) calculated for 1000 replications. The tree was rooted with *Sulfolobus solfataricus* as an outgroup.

The ML phylogenetic tree inferred from the 16s rRNA sequences revealed four distinct subgroups of bacterial isolates: *Brevundimonas*, *Leucobacter*, *Alcaligenes*, and *Staphylococcus*. In the *Brevundimonas* clade, strains CN004, CN006, CN007, and CN010 were clustered together with *B. naejangsanensis* (NR_116722), and the clade was supported with a strong bootstrap value of 100%. Also, our phylogenetic analysis showed that the *Alcaligenes* and *Staphylococcus* subgroups form a sister clade to the *Brevundimonas* subgroup. Furthermore, the CN008 and CN009 strains were placed phylogenetically close to *L. aridicollis* (NR_042288) within the *Leucobacter* clade. The high bootstrap value (100%) for the *Leucobacter* clade supports the robustness of this analysis (Figure 12).

## 4. Discussion

A collection of 120 rhizobacteria was isolated from argan rhizospheric and examined for their performances. The selected isolates showed in vitro positive PGPR traits, such as phosphate solubilization and IAA production. Furthermore, PGPR application led to a significant increase in germination-related parameters compared to the control. The isolate, *Leucobacter aridicollis* sp2, significantly increased radicle length with a value of 2.71 cm over the control and raised the germination rate by 95.83%. Improvement in this parameter was observed in other isolates, such as *Brevundimonas naejangsanensis* sp3, with a value of 2.42 cm. These results were similar to those reported by [13], who found that the *Pseudomonas* genus can increase the root length of wheat crops under saline conditions by up to 57% compared to the result obtained by the *Pseudomonas paralactis* strain, which increased the root length by 33% compared to the control. Moreover, [36] found that *B. megaterium* increased soybean germination by 3.3%, root length by 43.8%, root dry weight by 17.2%, and seedling vigor index by 32.4% compared to the control.

In addition, argan seedlings were inoculated under greenhouse conditions, and a significant increase was observed in height, stem diameter traits, and the number of ramifications. Among the four PGPR strains used under greenhouse conditions, the most effective bacteria for plant height were *Leucobacter aridicollis* sp2 and *Leucobacter aridicollis* sp1, increasing growth by 39.99 and 42.07%, respectively. Similarly, a significant increase in collar diameter was observed for the four isolates tested. The isolates, *Brevundimonas naejangsanensis* sp3 and *Leucobacter aridicollis* sp1, increased the collar diameter by 41.56% and 41.21%, respectively, followed by *Leucobacter aridicollis* sp2 (38.68%) and *Staphylococcus saprophyticus* (22.79%). For the ramification number, the isolate that significantly improved the number of branches was *Leucobacter aridicollis* sp1 (12) compared with the control plant (6). The four selected strains present various plant growth-promotion traits, such as nitrogen fixation, the ability to solubilize phosphate, siderophore production, and antagonistic activity against different phytopathogens. Similar results were observed when inoculated with *Leucobacter* sp., which significantly improved the fitness of tomato plants under well-watered and water-stressed conditions in terms of shoot height, stem diameter, and number of leaves [37], showing the highest nitrogenase activity (41.57 ± 0.57 µmol C_2_H_4_ h^−1^ mL^−1^) [38]. A similar improvement was observed in the growth of Bt-cotton inoculated with *Brevundimonas* sp. Its effect improved plant growth, as shown by the significant increase in plant height (68.41%), shoot dry weight (58.44%), and root dry weight (64.81%) compared to the untreated control [39]. A stimulatory effect was observed on chickpea plant growth by inoculation with the strain *Brevundimonas* sp., which resulted in a 7.40–26.21% increase in shoot height as compared to the control plants [40]. *Staphylococcus saprophyticus* has shown that it possesses the attributes of plant growth-promoting rhizobacteria such as phosphate solubilization, indole-3-acetic acid biosynthesis, siderophore production abilities, and biological nitrogen fixation [41].

Previous studies have shown that inoculation with different PGPR enhances the height of plant, such as the height of cucumber inoculated with rhizobacteria, including *Pseudomonas paralactis*, *Sinorhizobium meliloti*, and *Acinetobacter radioresistens*, and showed a significant increase compared to the control [42]. Inoculation with *S. meliloti* and *A. radioresistens* showed an improvement in stem diameter of 36 and 30%, respectively, and an improvement in dry biomass of 59 and 83%, respectively. Regarding root length, *A. radioresistens* promoted an increase of 135%. Similar enhancement of seed germination parameters has been reported across various cereals, including sorghum and pearl millet, by all isolates of *Pseudomonas fluorescens*, with a 92% improvement [43]. The PGPR-induced improvement in seed germination has also been observed in the cases of wheat and sunflower. Some PGPR induced an increase in seed emergence and achieved up to 100% more than the controls [44]. These results may be attributed to the increased synthesis of certain essential hormones such as auxins, gibberellins, ethylene, cytokinins, and abscisic acid, all of which are significant for plant growth and development.

Bacterial inoculants can increase plant growth and germination rates, improve seedling emergence and responses to external stress factors, and protect plants from disease. The current work revealed that under in vitro conditions, seed treatment with PGPR isolates improved rate germination and radicle length compared to the control. Greenhouse experiments revealed that all four strains had a positive effect on the growth of the argan tree, in particular *Leucobacter aridicollis* sp1, which, in addition to P solubilization and IAA production, nitrogen fixation is one of the most effective and environmentally friendly mechanisms for increasing plant growth [45]. Nitrogen is the most vital nutrient for plant growth and productivity. Although there is approximately 78% N_2_ in the atmosphere that cannot be used in this inert form, plants have a nitrogenase enzyme used to convert nitrogen into ammonia, which is the form that plants can use [46].

It will be necessary to conduct further research to elucidate how PGPR promote plant growth and optimize their application in agriculture. Understanding these mechanisms is essential for developing effective approaches to improve crop productivity, reduce the impact on the environment, and maintain food security for the future.

## 5. Conclusions

This study tried to expose the rhizosphere of the argan tree and, more precisely, the bacteria that could both solubilize phosphate, produce auxin, and give important results during inoculation for different plants. This study concluded that the utilization of native PGPR either alone or as bioinoculants is particularly pertinent when discussing the *Leucobacter aridicollis* sp1 strains, which improve *Argania spinosa* rate germination and radicle length in vitro, and height, stem diameter, and ramifications under greenhouse conditions. Seed, soil, and plant inoculations with PGPR are a promising approach to improving global agricultural production and optimizing nutrient use efficiency. In the present study, we highlight that the plant rhizosphere is a valuable source of potent rhizobacteria that can serve as an ecological solution for biofertilization by different mechanisms without compromising the safety of the environment. In the future, it will be necessary to study the effects of bacterial inoculation on the productivity and oil quality of the argan tree. In conclusion, the results of this study suggest that simultaneous screening of rhizobacteria for growth and yield promotion under pot and field experiments is an efficient tool to select effective PGPR for biofertilizer development and biotechnology.

## Figures and Tables

**Table 1:** Estimation of the qualitative potential of PGPR isolates in solubilization halo (cm) and IAA production.

Isolates	P-Solubilisation Halo (cm)	IAA Production	Isolates	P-Solubilisation Halo (cm)	IAA Production
CN001	1.40 ± 0.00	+	CN083	0.86 ± 0.09	−
CN002	1.63 ± 0.12	+	CN099	0.90 ± 0.00	−
CN003	0.56 ± 0.14	+	CN076	0.96 ± 0.05	−
CN004	1.87 ± 0.05	+	CN061	1.07 ± 0.09	−
CN005	1.33 ± 0.17	+	CN118	1.07 ± 0.17	−
CN006	1.43 ± 0.05	+	CN101	1.10 ± 0.14	−
CN007	1.83 ± 0.12	+	CN120	1.13 ± 0.09	−
CN008	1.47 ± 0.17	+	CN119	1.17 ± 0.24	−
CN009	2.23 ± 0.05	+	CN074	1.20 ± 0.08	−
CN010	2.90 ± 0.08	+	CN114	1.23 ± 0.05	−
CN011	1.40 ± 0.00	+	CN088	1.27 ± 0.05	−
CN012	1.27 ± 0.05	+	CN097	1.30 ± 0.08	−
CN013	1.40 ± 0.08	+	CN102	1.33 ± 0.05	−
CN014	1.30 ± 0.00	+	CN100	1.37 ± 0.05	−
CN015	1.73 ± 0.09	+	CN064	1.40 ± 0.08	−
CN016	1.27 ± 0.12	+	CN098	1.47 ± 0.05	−
CN017	0.87 ± 0.09	+	CN089	1.53 ± 0.05	−
CN018	2.00 ± 0.16	+	CN071	1.55 ± 0.74	−
CN019	2.00 ± 0.00	+	CN108	1.67 ± 0.24	−
CN020	1.90 ± 0.00	+	CN086	1.80 ± 0.00	−
CN021	1.40 ± 0.08	+	CN052	1.86 ± 0.05	−
CN022	1.15 ± 0.05	+	CN080	1.90 ± 0.90	−
CN023	1.30 ± 0.08	+	CN069	2.00 ± 0.24	−
CN024	2.00 ± 0.16	+	CN115	2.03 ± 0.05	−
CN025	1.10 ± 0.00	+	CN109	2.83 ± 0.12	−
CN096	0.80 ± 0.08	−			
CN029	0.83 ± 0.17	−			

Note: The values are presented as mean ± standard error, positive (+), negative (−).

**Table 2:** PGPR parameters.

Strains	Strains	P Solubilization (µg/mL)	IAA Production (µg/mL)	HCN Production	N_2_ Fixation	Ammonia Production	Siderophore Production
CN011	*Staphyloccocus saprophyticus*	49.11 ± 0.76	137.14 ± 0.33	−	−	+	+
CN006	*Brevundimonas naejangsanensis* sp3	36.21 ± 0.11	27.83 ± 0.49	−	−	+	+
CN009	*Leucobacter aridicollis* sp2	42.30 ± 0.77	97.47 ± 1.65	+	−	+	+
CN008	*Leucobacter aridicollis* sp1	41.96 ± 0.89	60.29 ± 4.66	−	+	+	+

Note: The values are presented as mean ± standard error, positive (+), negative (−).

**Table 3:** Morphological, biochemical, and physiological characteristics of bacteria.

Isolates		CN011	CN006	CN009	CN008
Strains		*Staphyloccocus saprophyticus*	*Brevundimonas naejangsanensis* sp3	*Leucobacter aridicollis* sp2	*Leucobacter aridicollis* sp1
Colony color		Yellow	White	White	White
Colony texture		Smooth	Smooth	Smooth	Smooth
Gram stain		−	+	+	+
Catalase		+	+	+	+
Oxidase		+	−	−	−
Motility		−	−	−	−
Shape		Cocci	Bacilli	Bacilli	Bacilli
Temperature	4 °C	−	+	+	−
	10 °C	−	+	+	+
	30 °C	+	+	+	+
	55 °C	+	−	−	−
NaCl	5 g/L	+	+	+	+
	10 g/L	+	+	+	+
	20 g/L	+	+	+	+
	40 g/L	+	+	+	−
pH	4	+	+	+	+
	5	+	+	+	+
	7	+	+	+	+
	10	+	+	+	+
	12	+	+	+	+

Note: The values are presented as mean ± standard error, positive (+), negative (−).

## Data Availability

All data generated in this work are provided within this manuscript.
